# A Synchrotron‐Based Study of the *Mary Rose* Iron Cannonballs

**DOI:** 10.1002/anie.201713120

**Published:** 2018-03-08

**Authors:** Hayley Simon, Giannantonio Cibin, Phil Robbins, Sarah Day, Chiu Tang, Ian Freestone, Eleanor Schofield

**Affiliations:** ^1^ The Mary Rose Trust College Road, HM Naval Base Portsmouth PO1 3LX UK; ^2^ Institute of Archaeology University College London (UCL) 31–34 Gordon Square London WC1H 0PY UK; ^3^ Diamond Light Source Harwell Campus Didcot Oxfordshire OX11 0DE UK

**Keywords:** archaeology, chlorine, corrosion, iron, X-ray absorption spectroscopy

## Abstract

Post‐excavation iron corrosion may be accelerated by the presence of Cl^−^, leading to conservation methods designed to remove Cl. This study exploits a unique opportunity to assess 35 years of conservation applied to cast‐iron cannon shot excavated from the Mary Rose. A combination of synchrotron X‐ray powder diffraction (SXPD), absorption spectroscopy (XAS), and fluorescence (XRF) mapping have been used to characterise the impact of conservation on the crystalline corrosion products, chlorine distribution, and speciation. The chlorinated phase akaganeite, β‐FeO(OH,Cl), was found on shot washed in corrosion inhibitor Hostacor IT with or without an additional reduction stage. No chlorinated phases were observed on the surface of shot stored in sodium sesquicarbonate (Na_2_CO_3_/NaHCO_3_); however, hibbingite, β‐Fe_2_(OH)_3_Cl, was present in metal pores. It is proposed that surface β‐FeO(OH,Cl) formed in the early stages of active conservation owing to oxidation of β‐Fe_2_(OH)_3_Cl at near‐neutral pH.

For over 2000 years, iron has been used to manufacture weapons, tools, ceremonial items, and more. However, surviving artefacts are prone to permanent loss or damage through the action of corrosion. During burial, the metal oxidises by an electrochemical process, where the cathodic reaction and oxidising agent are dependent on environmental conditions.^1^ Commonly, this involves either H_2_ evolution or O_2_ reduction,^2^ with multiple pathways involved in the reaction, resulting in complex corrosion layers.^3^, ^4^, ^5^ Under favourable conditions, the corrosion rate may be sufficiently low to allow exceptional preservation of artefacts. One such case is the shipwreck of King Henry VIII's flagship, the *Mary Rose* (1511–1545), which sunk off the coast of Portsmouth on July 19, 1545. Buried in sediment 14 m below the surface,^6^ the ship was held in an environment with dissolved O_2_ concentration of 0 mg L^−1^ and redox potential, *E_h_*, between −34 and −110 mV.^6^ These conditions allowed the ship and ca. 19 000 artefacts to survive until excavation between 1979 and 1982.

Iron objects from sites with good preservation conditions face a greater threat from corrosion that occurs after excavation. Exposure to air and water can cause oxidation of Fe^0^ to stable Fe^II^, Fe^III^, and intermediate Fe^II/III^ compounds,^1^ leading to rapid deterioration (Figure 1 a). Furthermore, in the presence of Cl^−^, the reaction rate increases,^7^ resulting in preservation challenges for objects buried in chlorine‐rich environments, such as seawater.^7^, ^8^, ^9^, ^10^ To mitigate this, several desalination treatments have been proposed that aim to remove as much Cl as possible from the artefact. These techniques can be divided into two categories: reduction‐based,^11^, ^12^, ^13^ where Cl^−^ is removed by transformation of chorine‐containing Fe^III^ crystals, and washing methods^10^, ^14^ that remove chlorine by diffusion into aqueous solution.^15^ Comparison of different conservation methods^10^, ^16^, ^17^, ^18^ has been limited by the variability of both objects and burial environments. As a result, it is often not possible to attribute differences in treatment success to technique used, while studying material that accurately reflects an archaeological artefact. To overcome these issues, this work focuses on the collection of 1248 cast iron cannon shot from the *Mary Rose*.

**Figure 1:**
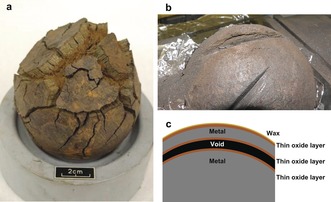
Mechanical damage to *Mary Rose* cast iron cannon shot: a) shot showing severe cracking and degradation in storage [photo credit: The *Mary Rose* Trust], b) typical structure observed during artefact sampling, as represented in c) with a very thin layer of surface corrosion and separation of original metal into two layers owing to internal voids and cracks.

Having been produced in bulk,^19^ the shot were buried together and their relative uniformity maintained until excavation, when they were treated by a number of conservation methods and exposed to varying environmental conditions.^20^ Immediately after excavation, all of the shot were immersed in a high pH solution (either NH_3_, NaOH, or an equimolar mixture of Na_2_CO_3_/NaHCO_3_) until undergoing active conservation, though some shot have remained in passive storage to the present day. For this study, three differently treated categories of shot have been investigated:

1. SS: passive storage in 0.15 m (pH 10) sodium sesquicarbonate, Na_2_CO_3_/NaHCO_3_, solution
2. HW: same as (1) until 2010–12, when rinsed in tap water, washed in 3 or 4 consecutive baths of 1 % *v:v* corrosion inhibitor Hostacor IT:water,^21^ (Scheme 1), pH 4.5–8.5, and dried in 2‐stage acetone:water (1:1 and 1:0) series. Now stored in a controlled environment (20 °C, 20 % RH)
  **Scheme 1:**
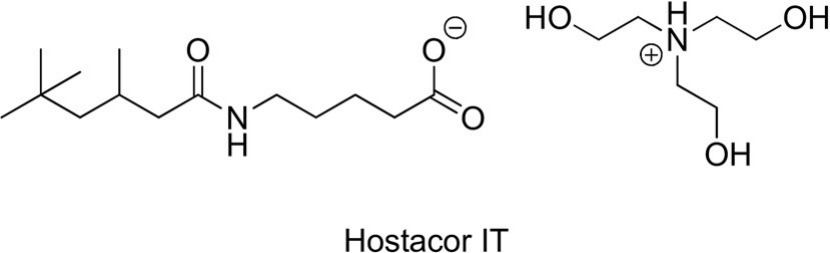
Structure of corrosion inhibitor Hostacor IT, a corrosion inhibitor for iron in polyethylene glycol solution, used in treatments HW and HWAS.
3. HWAS: same as (2) but underwent additional alkaline sulfide, NaOH/Na_2_SO_4_, pH 12–13 reduction treatment.^13^ Now stored in controlled (20 °C, 20 % RH) environment

Two types of samples were collected: bulk corrosion powders from the object surface (**‐C** samples) and cut cross‐sections mounted in polyester resin (**‐S** samples; Table 1). Crystalline phases were studied by SXPD of **‐C** samples, while the chlorine distribution and speciation of **‐S** samples were probed by synchrotron XRF mapping and Cl k‐edge X‐ray absorption near‐edge spectroscopy (XANES), respectively. Details of the method may be found in the Supporting Information.

**Table 1:** Details of samples and shot analysed in this study.

Shot ID	Dimensions		Treatment	No. of samples analysed	
	*d* [mm]	Weight [g]		C	S
81A1527	85.0	2410.8	SS	2	0
81A2310	82.6	2199.9	SS	2	0
81A3470	84.8	2107.0	SS	2	0
81A3550	84.1	2361.0	SS	1	0
81A3839	86.0	2014.8	SS	3	1
82A2618	85.3	2012.5	SS	4	1
82A4365^[a]^	99.0	3500.0	SS	1	0
82A4233^[a]^	86.0	2802.6	SS	2	0
81A3461^[a]^	84.0	568.3	SS	2	0
81A6218^[a]^	193.0	26 000	SS	1	0
81A6219^[a]^	192.0	25 750	SS	1	0
81A6143	79.9	1818.1	HWAS	1	1
83A0161	83.6	1743.2	HW	1	1
83A0189	70.6	1263.7	HWAS	1	1
83A0446	^[b]^	1052.0	HW	1	0
81A0177	^[b]^	^[b]^	HWAS	1	0
81A6102	83.7	2045.1	HW	0	1

[a] Diameter, *d*, and weight measurements from excavation record. [b] Artefact in too many pieces for accurate measurement.

During cutting, cracks were observed in HW and HWAS shot, presenting a structure (Figure 1 b,c) that consists of a thin (<0.5 mm) surface corrosion layer over the original Tudor metal. Corrosion around large cracks and casting voids is associated with severe degradation and mechanical failure. In some cases, this has led to fracturing, indicating that corrosion is occurring inside the artefact, rather than from the outside in.

The chlorinated phase most often associated with archaeological iron corrosion in chlorinated media is akaganeite,^22^, ^23^, ^24^ commonly referred to as β‐FeO(OH), though more closely defined as FeO_0.833_(OH)_1.167_Cl_0.167_.^22^ Akaganeite has a monoclinic crystal structure (space group: *I*2/*m*), built from edge‐ and corner‐sharing Fe(O,OH)_6_ octahedra that form a tunnel structure similar to the hollandite crystal.^22^, ^25^, ^26^ Two Cl sites may be distinguished:^23^ within the tunnel structure, *Cl_str_*, and adsorbed to the surface, *Cl_sur_* of the crystal, Figure 4 b. The size of individual β‐FeO(OH) crystals depends on the growth conditions, but is typically small, around 0.15×0.03 μm, resulting in a large proportion of *Cl_sur_*.^22^ During a washing‐based conservation treatment, mobile *Cl_sur_* is removed, while *Cl_str_* is thought to be unaffected.^14^, ^22^, ^23^, ^27^ X‐ray diffraction of akaganeite gives two low‐angle peaks, which at the wavelength used in this study (*λ*=0.82578 Å) appear at 2*θ* values 6.35° and 8.95°, corresponding to the (101) and (200) planes, respectively. The insets in Figure 2 a highlight the location of these peaks (dotted grey lines) and show that, somewhat surprisingly, akaganeite is not observed in the surface corrosion products of any of the SS shot, while it is present on the surface of all HW and HWAS shot. Phase identification of the powder diffraction profiles, Figure 2 b, instead shows a combination of phases from the burial environment: calcite (CaCO_3_), quartz (SiO_2_), and aragonite (CaCO_3_); related to the microstructure of the metal: graphite and cementite (Fe_3_C); and commonly reported^3^, ^14^ marine iron corrosion products: goethite α‐FeOOH, magnetite Fe_3_O_4_ and lepidocrocite γ‐FeOOH.

**Figure 2:**
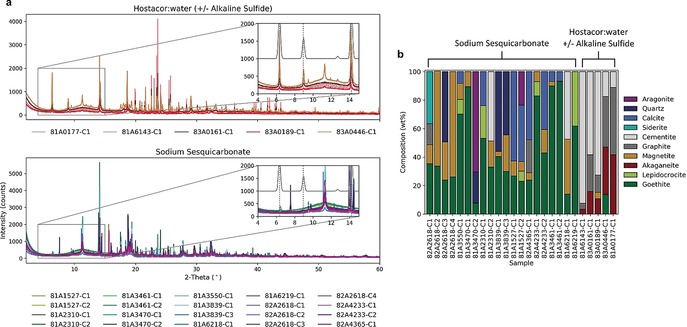
a) SXPD of corrosion powders from top: HW (brown) and HWAS (red) treated shot and bottom: SS (green–purple) passively stored shot. Insets show 1.5× zoom of region 4–15° 2*θ*, with ICSD database pattern for akaganeite (69 606)^26^ highlighted in grey. Dotted lines show the expected positions of akaganeite peaks at 6.35°, 8.95°, and 14.20° (*λ*=0.82578 Å). b) Phases identified by the QUALX2^35^ software and semi‐quantitative phase proportions, based on the reference intensity ratio method,^36^ *I*/*I*_c_ (c=corundum) accuracy ±5 wt %.

Reflecting the SXPD results, where no chlorinated corrosion products were observed on the surface of SS shot, Cl elemental maps of **‐S** samples (Figure 3 and the Supporting Information, Figure S7) show no chlorine at the surface of SS shot, while a localised layer of Cl is present on the outer edges and around voids of HW and HWAS shot. However, in one SS shot, the example shown in Figure 3, an area of Cl in the inner region of the sample can be seen. Comparing the Cl k‐edge XANES in this inner area to the chlorine species observed on a HW treated shot (Figure 4) it can be seen that the Cl species is different. To identify the chlorine species present in the *Mary Rose* samples, a library of Cl standards was prepared by a combination of chemical purchases and synthesis (see the Supporting Information). Cl XANES from the standards were used to fingerprint spectral features in the sample datasets. The library of standards included a lab‐synthesised sample of hibbingite, β‐Fe_2_(OH)_3_Cl, a precursor to akageneite that has been observed on archaeological iron^3^, ^28^, ^29^ but rapidly oxidises^30^, ^31^ to akaganeite in storage^32^ or during conservation^14^ and a lab‐synthesised standard of β‐FeO(OH), prepared by hydrolysis of an Fe^3+^ chloride solution. To simulate the effect of a washing treatment, the akaganeite standard was immersed in 500 mL distilled H_2_O for 1 month at ambient conditions, with solution changes every 48–72 hours, giving a series of incrementally washed standards **AKA‐1** (0 washes) to **AKA‐8** (7 washes). Comparing the spectra from the HW and SS shot, it can be seen that the HW spectrum is consistent with β‐FeO(OH), Figure 4 c, while the spectrum from beneath the surface of the SS shot, Figure 4 d, is consistent with the precursor Fe^II^ chloride, β‐Fe_2_(OH)_3_Cl.

**Figure 3:**
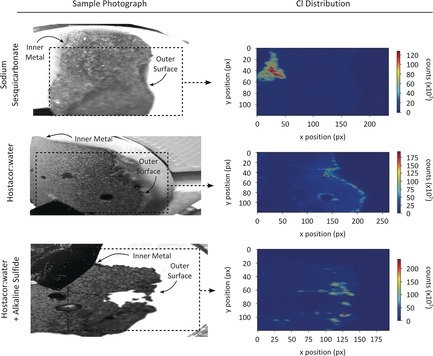
XRF elemental maps of the chlorine distribution in cut cross‐sections from SS, HW, and HWAS‐treated shot. Additional examples: Supporting Information, Figure S7.

**Figure 4:**
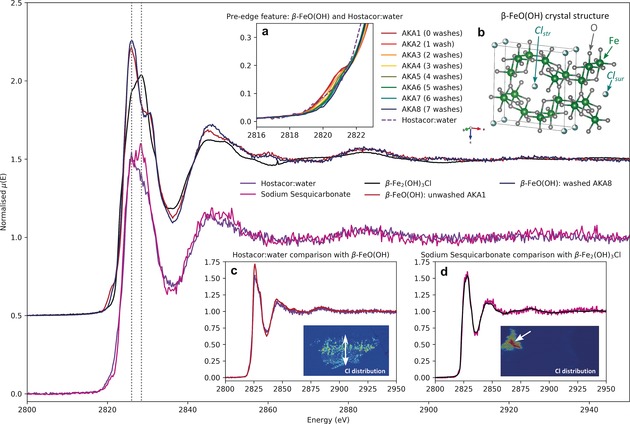
Cl k‐edge XANES of chlorine species observed on a HW treated shot (purple) and under the surface of an SS treated shot (pink) compared to standard spectra for washed and unwashed akaganeite, β‐FeO(OH) and hibbingite, β‐Fe_2_(OH)_3_Cl. Dotted grey lines show the position where the peak of the white line occurs for akaganeite (2826.02 eV) and hibbingite (2828.46 eV). Non‐offset comparisons of standards and sample spectra are shown in insets (a), (c), and (d) focusing on the pre‐edge region, HW, and SS respectively. Insets (c) and (d) additionally include XRF Cl element maps of the sample with white arrows indicating the location of XAS acquisition. Inset (b) shows the crystal structure of akaganeite, 1 unit cell wide in the *b* and *c* axis and 1.5 along the *a* axis.^26^

Looking at the pre‐edge region (Figure 4 a), a small feature is visible at 2819–2822 eV for the unwashed akaganeite that is decreased in amplitude for the washed standard. The XANES data from the series of washed standards, **AKA1**–**8**, were fitted with three peaks to compare the relative intensities of the observed features (Figure 5). While the contribution from the second and third peaks remains constant throughout the series (Figure 5 c, squares), the contribution from the first peak, the pre‐edge, decreases with an increasing number of washes (circles and stars). This feature arises from electronic transitions in partially‐bound *Cl_sur_*,^33^ demonstrating that chlorine is lost from this site during washing. For the HW sample, a reduced amplitude is observed, indicating that the phase has gone through several washing stages to remove *Cl_sur_*. From this, it may be inferred that β‐FeO(OH) formed prior to, or in the early stages of the washing treatment. Immediately before the HW shot underwent active conservation, it was stored at pH 10 and was analogous to SS shot, that is, no surface β‐FeO(OH), but sub‐surface chlorine present in pores as β‐Fe_2_(OH)_3_Cl. Studies have shown that oxidation of hibbingite to akaganeite in solution is only thermodynamically feasible in the pH range 4–6,^30^ and that complete transformation can occur in about 7 hours.^30^ As a result, it is proposed that the sudden change in pH from 10 to near‐neutral during active conservation, coupled with removal in tap water of adhering corrosion layers, led to exposure of previously‐blocked pores and cracks, enabling mobilisation of sub‐surface chlorine and subsequent oxidation to akaganeite at the object surface. This transformation would have occurred while the artefact was in the first washing bath, resulting in loss of *Cl_sur_* in successive washes. This conclusion is supported by a recent in situ study^34^ of hibbingite on archaeological iron, where akaganeite was not observed to form in NaOH, but was observed after drying at the end of the treatment. However, while this mechanism can be used to explain akaganeite formation on this sample, it only represents a single location on a single artefact. In cases where fracturing has occurred to an object and a new surface is exposed to the external environment, corrosion products can be observed^24^ to form while in storage or on display. Rather, this investigation has shown that, alongside its formation after conservation, there are additional opportunities for akaganeite to form on archaeological iron, such as during or in‐between treatment stages.

**Figure 5:**
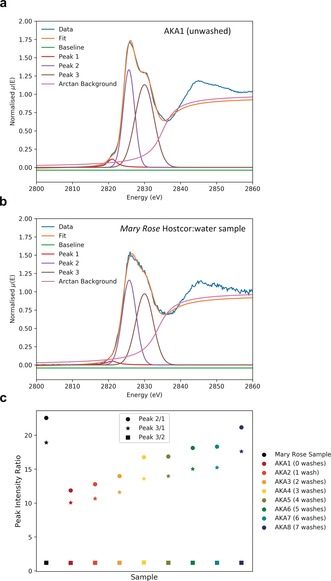
Results of 3‐peak fit to Cl k‐edge XANES for a) unwashed akaganeite standard and b) *Mary Rose* HW treated shot. c) Relative intensity contribution of each peak for the sample and washing series (Additional examples: Supporting Information, Figure S8).

In conclusion, this work has used a combination of synchrotron techniques to gain an unprecedented insight into the effect of conservation choices on iron corrosion. It has been shown that during multi‐decade immersion in sodium sesquicarbonate solution, chlorine is removed from the outer surface of artefacts; however, Cl can remain trapped in pores within the metal, in the form of hibbingite, β‐Fe_2_(OH)_3_Cl. On exposure to oxygen at near‐neutral pH, this phase rapidly oxidises to β‐FeO(OH). In the case of the *Mary Rose* shot, it is proposed that this transformation occurred during treatment, owing to a combination of pH change and surface removal. Using chlorine XANES spectra, it has been shown that, years after conservation, it is possible to differentiate iron corrosion products formed during treatment. This reveals that post‐conservation studies of artefacts, even decades after treatment, have an important role to play in the future development of conservation.

## Conflict of interest

The authors declare no conflict of interest.

## Supporting information

As a service to our authors and readers, this journal provides supporting information supplied by the authors. Such materials are peer reviewed and may be re‐organized for online delivery, but are not copy‐edited or typeset. Technical support issues arising from supporting information (other than missing files) should be addressed to the authors.

Supplementary
