# Supplementary material for: A Synchrotron‐Based Study of the Mary Rose Iron Cannonballs
Source: Angew Chem Int Ed Engl. 2018 Mar 8;57(25):7390–5. doi: 10.1002/anie.201713120 (PMC6032935; doi:10.1002/anie.201713120)
Supplement: Supplementary file 1 — Supplementary [file ANIE-57-7390-s001.pdf]

Angewandte Chemie
Eine Zeitschrift der Gesellschaft Deutscher Chemiker

Supporting Information

A Synchrotron-Based Study of the *Mary Rose* Iron Cannonballs

Hayley Simon, Giannantonio Cibin, Phil Robbins, Sarah Day, Chiu Tang, Ian Freestone, and Eleanor Schofield**

anie_201713120_sm_miscellaneous_information.pdf

Supporting Information

Experimental Methodology 2
Sampling Procedure 2
Synthesis of Standard Compounds 2
Synchrotron X-ray Powder Diffraction (SXPD) 3
X-ray Absorption Spectroscopy (XAS) 3
X-ray Fluorescence (XRF) Mapping 4
Table S1: XRF mapping area details 5
Fig. S1: 9 month sample powder XRD comparison 6
Fig. S2: Akaganeite standard SXPD 7
Fig. S3: Beamline set-up (B18) 8
Fig. S4: Cl XANES across HW sample 9
Fig. S5: TEY/Fluorescence comparison 10
Fig. S6: Additional Cl XANES standards 11
Fig. S7: Additional Cl XRF element maps 12
Fig. S8: Additional peak-fit results 13
Fig. S9: Weight change since excavation 14
SI References 15

Experimental Methodology

Synchrotron experiments were carried out at Diamond Light Source (Oxfordshire, UK) using the core-EXAFS beamline, B18, for XAS/XRF and the high resolution powder diffraction beamline, I11, for SXPD. Of the 17 shot studied in this investigation, 7 were sampled on-site at the *Mary Rose* conservation facility and have been marked by an [a] or [b] in Table 1 in the main document. The remaining 10 shot were transported to either UCL or Diamond for in-lab sampling. Before undertaking destructive sampling, the shot were weighed, measured with a digital calliper and photographed.

Sampling Procedure

Visual inspection of the artefacts informed the selection of locations for surface powder removal (**-C** samples). Here, the corrosion products were carefully cut away from the surface using a stainless steel scalpel, ground to a fine powder in an Agate pestle and mortar and transferred to a clean, dry Eppendorf tube. A comparison of the diffraction patterns collected immediately after sampling and following 9 months storage, Fig. S1, using a benchtop Rigaku MiniFlex 600 diffractometer (Rigaku, Sevenoaks, Kent) showed no change in the profile over this period. As a result, the powder samples were stored in a dry environment for up to 9 months prior to SXPD and XAS analysis.

Cross-sections (**-S** samples) were manually removed from the artefact using a hacksaw fitted with a stainless steel blade, 24 or 18 teeth per inch (tpi). Segments were cut into individual samples and mounted in a 2-part polyester resin† (MetPrep, Coventry, UK) as 25 mm circular blocks. The surface of the cross-sections were covered by a layer of resin until arrival at the beamline, where the outer layer was removed by grinding dry with a series of SiC abrasive discs (grades 240, 400, 800 and 1200). Between grades samples were dusted with compressed N_2 and on completion of final grade, the exposed sample was placed in Tupperware box flushed with N_2 prior to mounting in a holder on the beamline Fig. S3-B.

Synthesis of Standard Compounds

Materials

$FeCl_3$.6H2O, KCl, NaCl and NaOH were purchased from Sigma Aldrich (Gillingham, UK) and $FeCl_2$ from Fisher Scientific (Leicestershire, UK). All aqueous solutions were prepared using distilled water, dH_2O, from an ELGA PureLab OptionQ system (High Wycombe, UK). Centrifugation was performed using a Thermo Scientific Heraeus Multifuge 3SRT.

Akaganeite, β-FeO(OH)

A series of akaganeite standards with varying Cl content was prepared following the protocols of Schwertmann and Cornell[1] and Reguer et al.[2] Briefly, a 0.1 M solution of $FeCl_3$ was prepared and heated in a closed vessel at 70 °C for 48 hours. The resulting precipitate – see Fig. S2 for SXPD of product from this methodology - was collected via centrifugation by spinning at 1,500 rpm for 20 mins at room temperature. Excess salt from the synthesis was removed by washing the resulting pellet in dH_2O using the same centrifuge parameters. 0.3 g of this product was collected, dried for 10 mins at 50°C and stored in a clean, dry Eppendorf tube [**AKA1**]. A sample of the supernatant was collected to use as a standard for aqueous Cl^-. The remaining product

† One segment from shot 82A26218 was mounted in a 2-part bisphenol a epichlorohydrin epoxy resin (MetPrep, Coventry, UK). As this resin contains chlorine, it was not used for any other samples.

(~2 g) was re-suspended in 500 mL dH_2O and washed repeatedly for 1 month, with solution changes every 48-72 hours. At each solution change, 0.05-0.1 g of product was collect by centrifugation (1,500 rpm, 20 mins), giving a series of washed products, [**AKA2 to 8**], covering the 1 month period of washing. An additional unwashed sample was prepared using the same procedure, but was allowed to air-dry to investigate the impact of heated drying on the XANES features.

Hibbingite, β-$Fe_2(OH)_3Cl$
Hibbingite was prepared based on the methodology of Refait et al.[3,4] Solutions of $FeCl_2$ (50 mL, 0.13 M) and NaOH (50 mL, 1 M) were deaerated by purging with Ar for 2 hours before being transferred to a glovebox. The two solutions were combined while stirring vigorously at room temperature. Ion concentrations inside the reaction vessel were as follows: $[Cl^-]$ = 1.3 M, $[Fe^{2+}]$ = 0.65 M, $[OH^-]$ = 0.5 M. The resulting white suspension was aged for 24 hours, collected by filtering under gravity and allowed to air-dry before being transferred to a glass vial for storage in the glovebox.

Additional chlorine standards of KCl, NaCl, $FeCl_2$ and $FeCl_3.6H_2O$ were purchased for measurement during the XAS experiment.

Synchrotron X-ray Powder Diffraction (SXPD)
Powder samples (**-C**) were mounted in 0.5 mm quartz capillaries (Capillary Tube Supplies Ltd, Cornwall, UK) in a brass capillary holder secured with a cyanoacrylate adhesive (RS Components Ltd., Corby, UK). Measurements were collected at λ = 0.82578 Å (calibrated with NIST SRM640c Si standard) using 5 multi-analysing crystal (MAC) detectors[5] at ambient temperature and pressure. Exposure time for each capillary sample was 1800 s. Phase identification was performed using the software QUALX2,[6] by comparing the experimental patterns to the crystallography open database (COD).

X-ray Absorption Spectroscopy (XAS)
Cross-section samples: resin blocks
Cross-section samples were mounted together in a custom 3D-printed sample holder, and the sample, detector and beam were enclosed in a plastic bag flushed with He, Fig. S3-A/B/C. The beamline optics used Cr coated collimating and focusing mirrors, a Si(111) monochromator and Ni coated harmonic rejection mirrors. The first ion chamber (I_0) was filled with He gas. The spot size of the beam was ~100 x 100 µm. Cl k-edge XANES spectra were collected in fluorescence mode using a 4 element Vortex Si drift detector in the energy range 2722-3200 eV with steps of 0.2 eV. For the HW sample shown in Fig. 4, a series of 10 spots were analysed going from the internal to external surface, with 3 repeats each, Fig. S4. For the SS sample shown in Fig. 4, 1 spot was analysed and 5 repeat spectra collected.

Standards
KCl, NaCl $FeCl_2$, $FeCl_3$, akageneite powders AKA1-8 and the unwashed/airdried akaganeite were spread as a thin layer on graphite tape and mounted in the B18 soft X-ray chamber (aka: the *Mary Rose* chamber), Fig. S3-D, filled with 35 mbar He. An electrical contact for total electron yield, TEY, was made with Cu wire and the standards were measured by both TEY and fluorescence. TEY signal was collected in drain current mode with 200 V polarisation applied to an extraction ring located upstream of the sample. The beamline optics for measurement of standards in the soft

X-ray vessel used Pt coated mirrors, a Si(111) monochromator and Pt coated harmonic rejection mirrors. Cl k-edge XANES spectra were collected in the range 2750-3000 eV with steps of 0.09 eV. 1 repeat measurement was taken for $FeCl_2$ and $FeCl_3$; 3 for KCl and NaCl; 6 for the air-dried akaganeite; and 9 repeats for AKA1-9 and the solution sample. The hibbingite sample was spread on graphite tape in the glovebox and transferred to the beamline under inert (Ar) gas before analysis in vacuum. Care was taken to ensure that the sample was not exposed to oxygen during transport and analysis. The same beamline and XAS parameters were used for hibbingite as described for the other Cl standards, with 7 repeat measurements collected by TEY. To analyse the aqueous Cl^- standard, a pocket was made from 8 μm Mylar and graphite tape, into which the solution sample was injected and the sample was measured by fluorescence using the previously described parameters.

<u>*Data analysis*</u>

All XAS data were processed using *Athena,* part of the *Demeter* software package.[7] For both samples and standards, repeat spectra were merged and E_0 chosen as the first peak in the first derivative. At the 10 positions measured on the HW sample, the Cl species was found to be the same, Fig. S4, and the 30 spectra merged to 1. Merged sample spectra were normalised and background subtracted using a 3rd order polynomial and normalisation range from 31 eV above E_0 to the end of the collected dataset. The quality of the background model was assessed using difference spectra throughout the normalisation process. For the standard spectra, comparison of the fluorescence data to the electron yield spectra, Fig. S5, was used to ensure that self-absorption effects were not present. In the main document, Fig. 4 shows the fluorescence spectra for the akaganeite washing series, which have been normalised using a 2nd order polynomial and normalisation range from 78 eV above E_0 to the end of the dataset.

To exclude the possibility that the pre-edge feature arises from the drying procedure or aqueous Cl, the akaganeite washing series was compared to the solution sample and an air-dried akaganeite, as shown in Fig. S6.

A 3-peak model was developed to represent the 3 features observed in the XANES spectra and applied to the experimental data to allow a comparison of the relative intensity of the pre-edge. Fitting was performed using a script written in *Python 3.6,* with peak 1, the pre-edge, modelled as a lorentzian curve and peaks 2 and 3 modelled as gaussians. The centroid positions of peaks 1 and 3 were fixed at 2821 and 2830 eV respectively, while the position of peak 2 and the intensity and width of all 3 peaks were allowed to vary. An arctangent function, with a jump of 1, was used to model the background. The fit was refined using the *minimise* function of the *SciPy* python package.

X-ray Fluorescence (XRF) Mapping

XRF maps were collected on **–S** samples, using the same set-up previously described for XAS and shown in Fig. S3-A/B/C. Fluorescence detection was performed with a 4 element Si drift detector at 7.1 keV across the following areas, steps and speed:

Table S1: XRF mapping area details

Sample ID	X distance (mm)	X step (mm)	Y distance (mm)	Y step (mm)	Acquisition speed (mm/s)	Treatment
81A6102-S2A	12.0	0.10	8.0	0.1	1.0	HW
83A0189-S3A	14.0	0.05	9.4	0.1	0.5	HWAS
83A0161-S3A	13.5	0.05	12	0.1	0.5	HW
81A6143-S1B	10.0	0.05	12	0.1	0.5	HWAS
81A3839-S1B	12.0	0.05	12	0.1	0.5	SS
82A2618-S8	20.0	0.1	20	0.1	1.0	SS

The raw map data was exported as a series of HDF5 files and processed into 2D element maps using in-house mapping software developed at the B18 beamline,[8] written in *Python 3.6*.

Fig. S1: 9 month sample powder XRD comparison

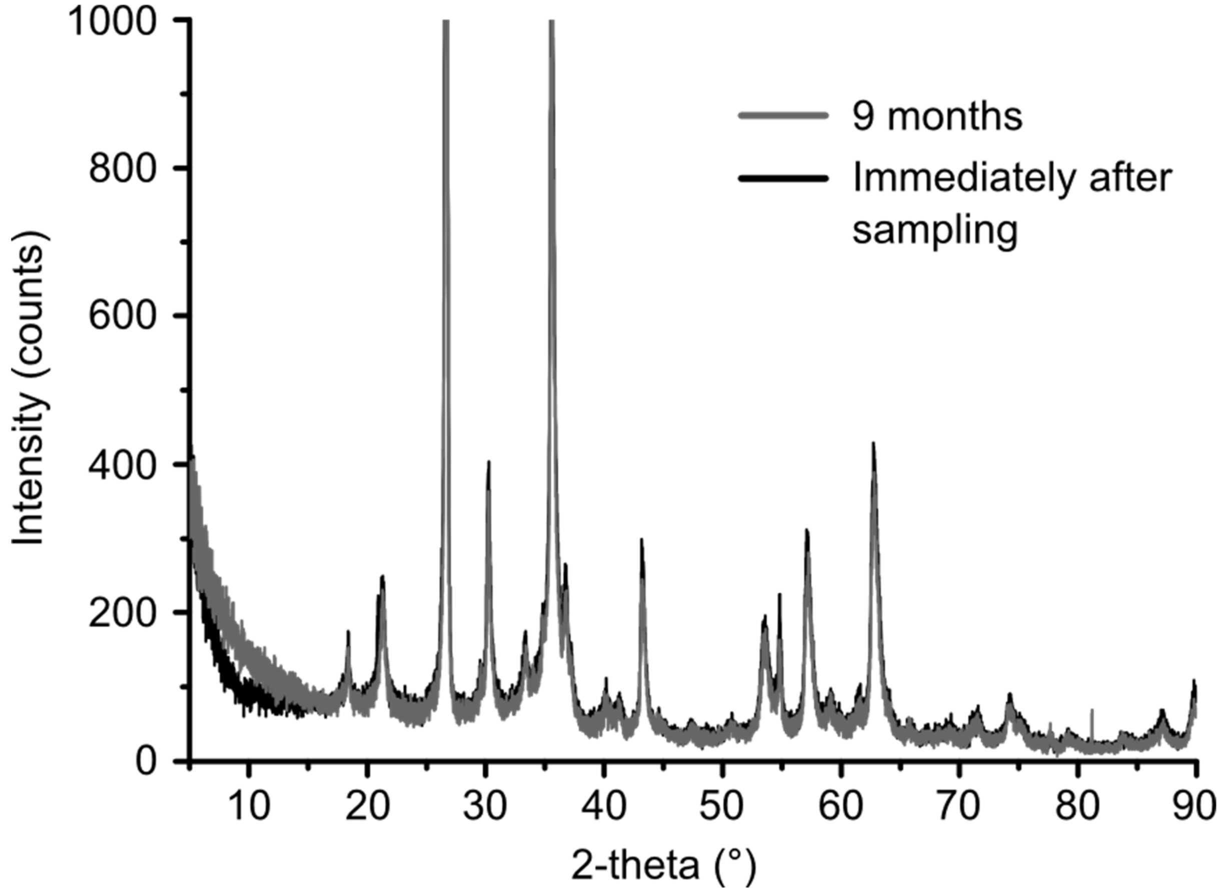

Fig. S1 Powder X-ray diffraction pattern of corrosion sample 82A2618-C2 taken immediately after sampling (black) and following 9 months in storage (grey). Measurements were performed on Rigaku MiniFlex 600 diffractometer with a copper target (Cu Kα, graphite monochromator), voltage 40 kV, current 15 mA, step size 0.0075°, duration 1.5s over the 2θ angular range 5-90º

Fig. S2: Akaganeite standard SXPD

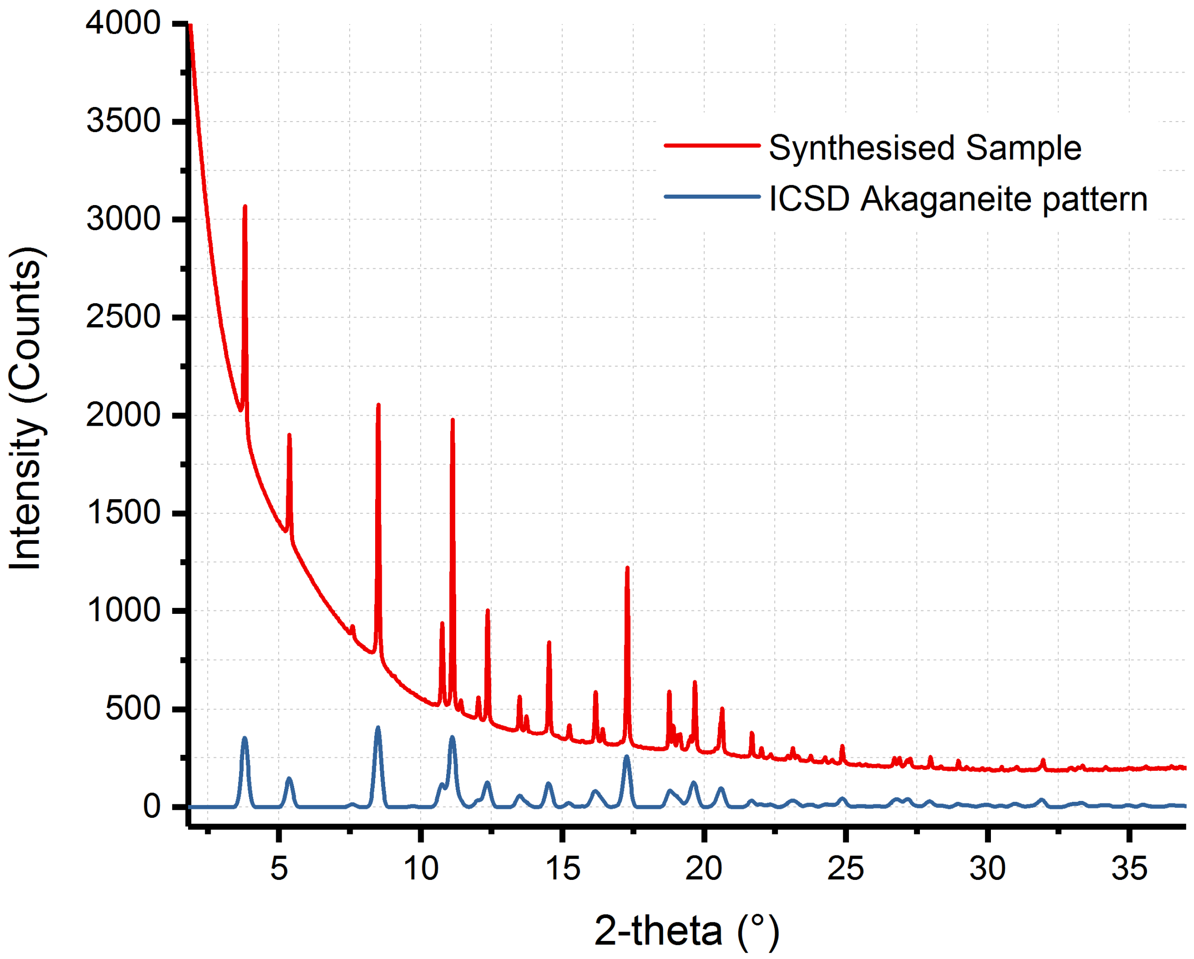

Fig. S2 SXPD of a sample of akaganeite synthesised using the methodology described above. Measurement was collected on beamline I11 experimental hutch 2 on a loose powder mounted in a washer using a Pixium digital area detector (RF4343, Thales) at λ = 0.494371 Å. For comparison, a simulated pattern of akaganeite is shown at the same wavelength (ICSD: 69606)

Fig. S3: Beamline set-up (B18)

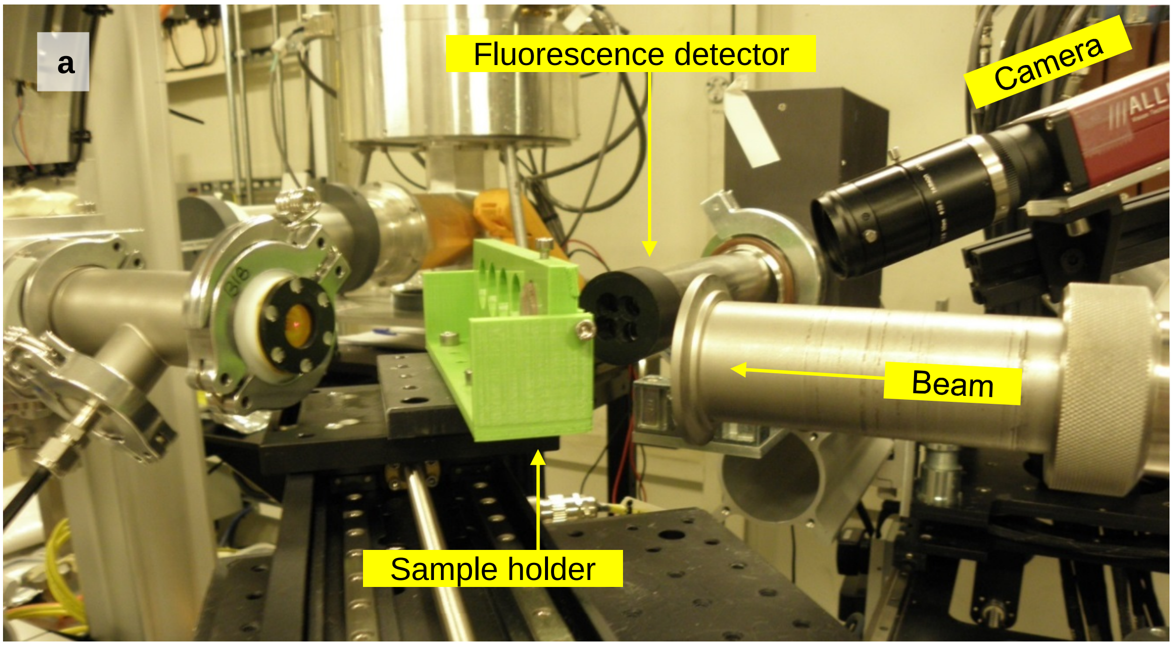

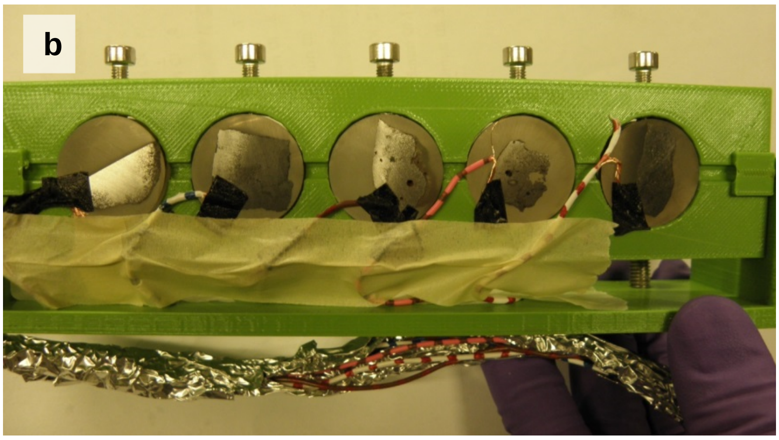

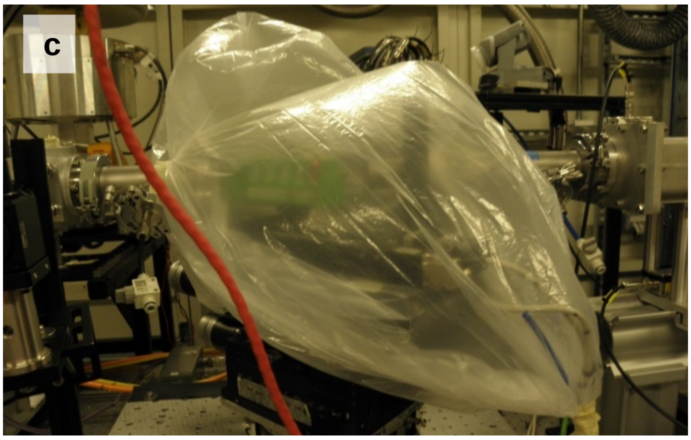

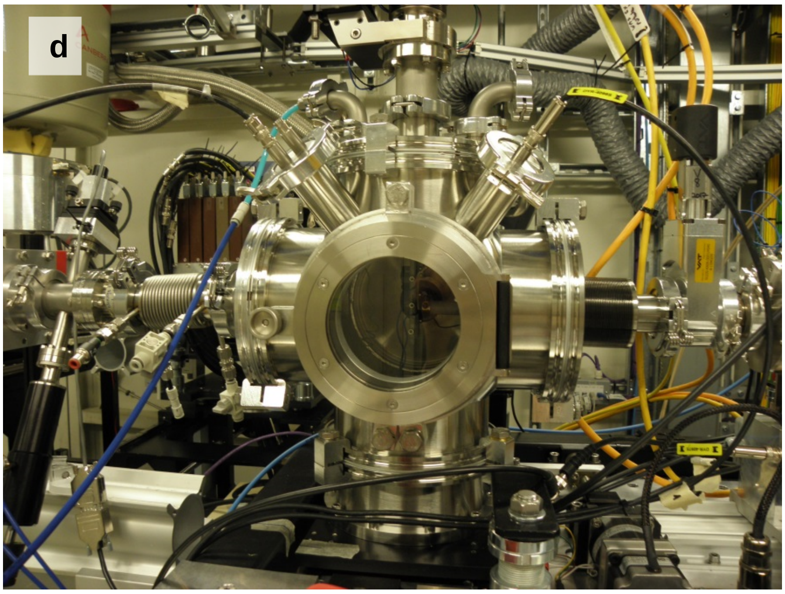

Fig. S3 Experimental set-up for XAS and XRF measurements on beamline B18, showing **a)** location of detectors and sample, **b)** sample holder used for mounted cross-sections (**–S** samples) and **c)** measurement conditions, with entire set-up enclosed in a plastic bag filled with He gas. Photo **d)** shows the soft X-ray sample chamber used to measure standards by TEY and fluorescence.

Fig. S4: Cl XANES across HW sample

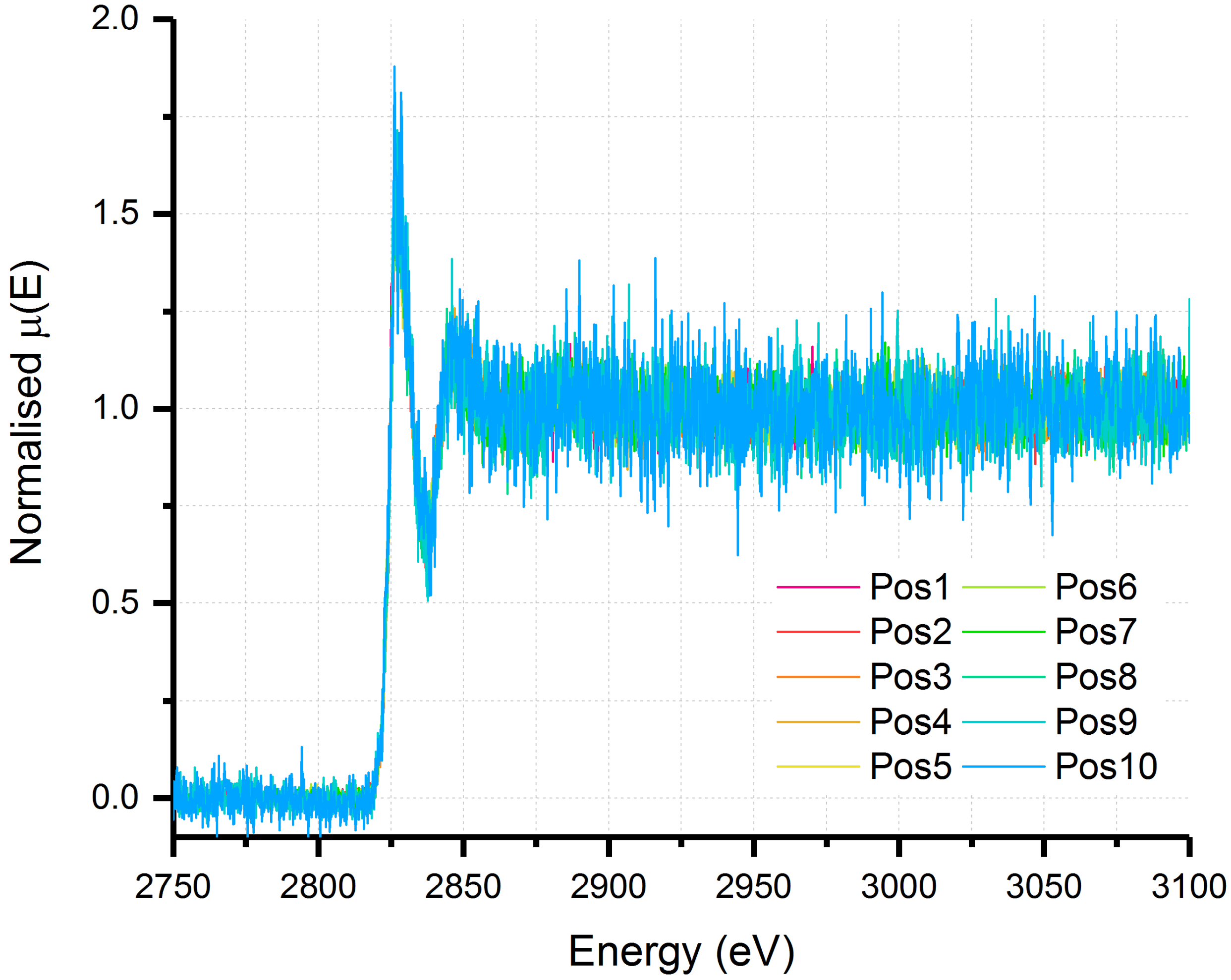

Fig. S4 Cl k-edge XAS spectra collected across 10 positions on the HW sample in main text Fig. 4, showing the same Cl-species across the whole sample (3 repeats per position)

Fig. S5: TEY/Fluorescence comparison

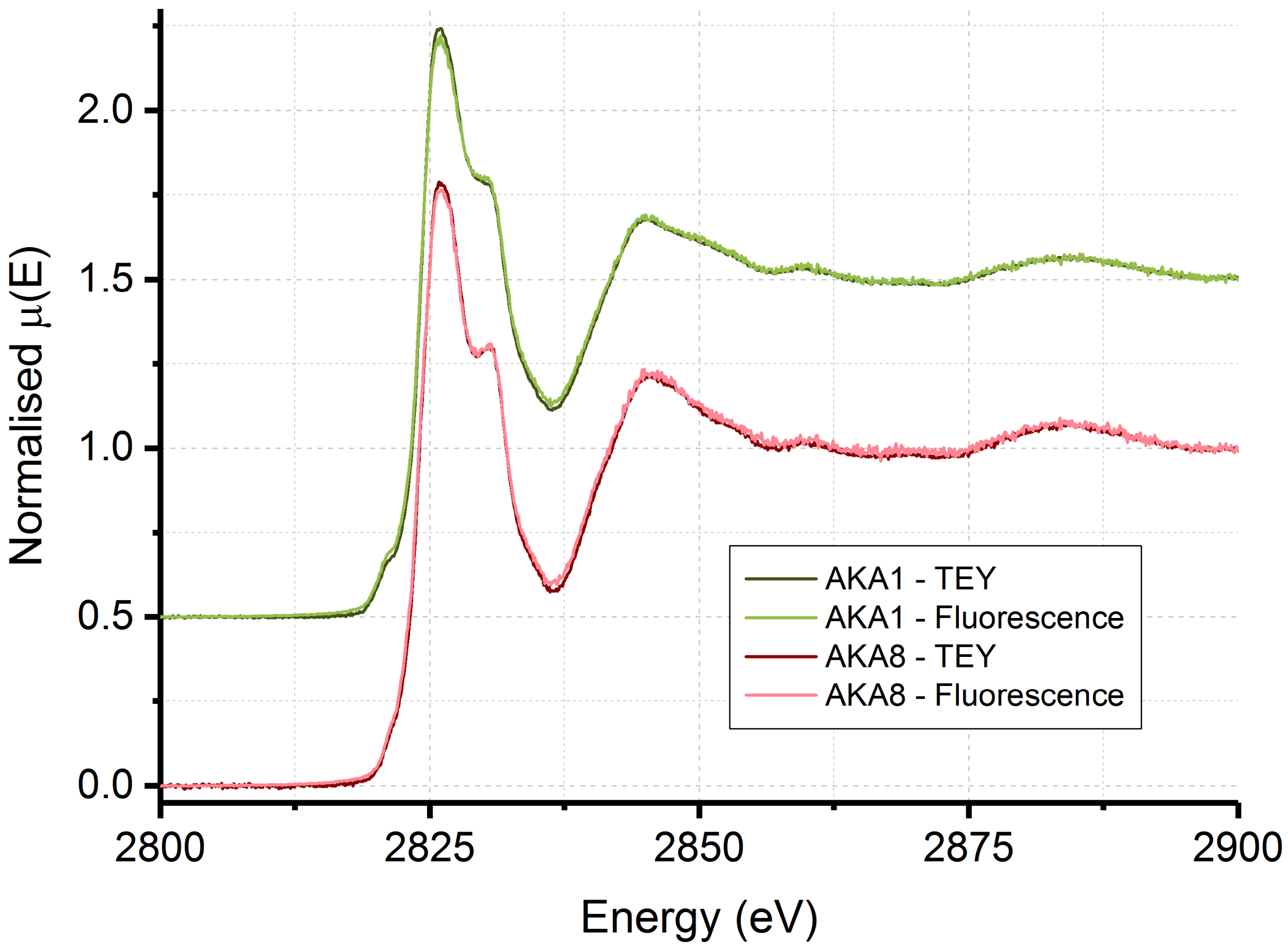

Fig. S5 Comparison of normalised XANES spectra for the first (**AKA1**) and last (**AKA8**) akaganeite wash samples collected by TEY and fluorescence, demonstrating no self-absorption effects across the washing series

Fig. S6: Additional Cl XANES standards

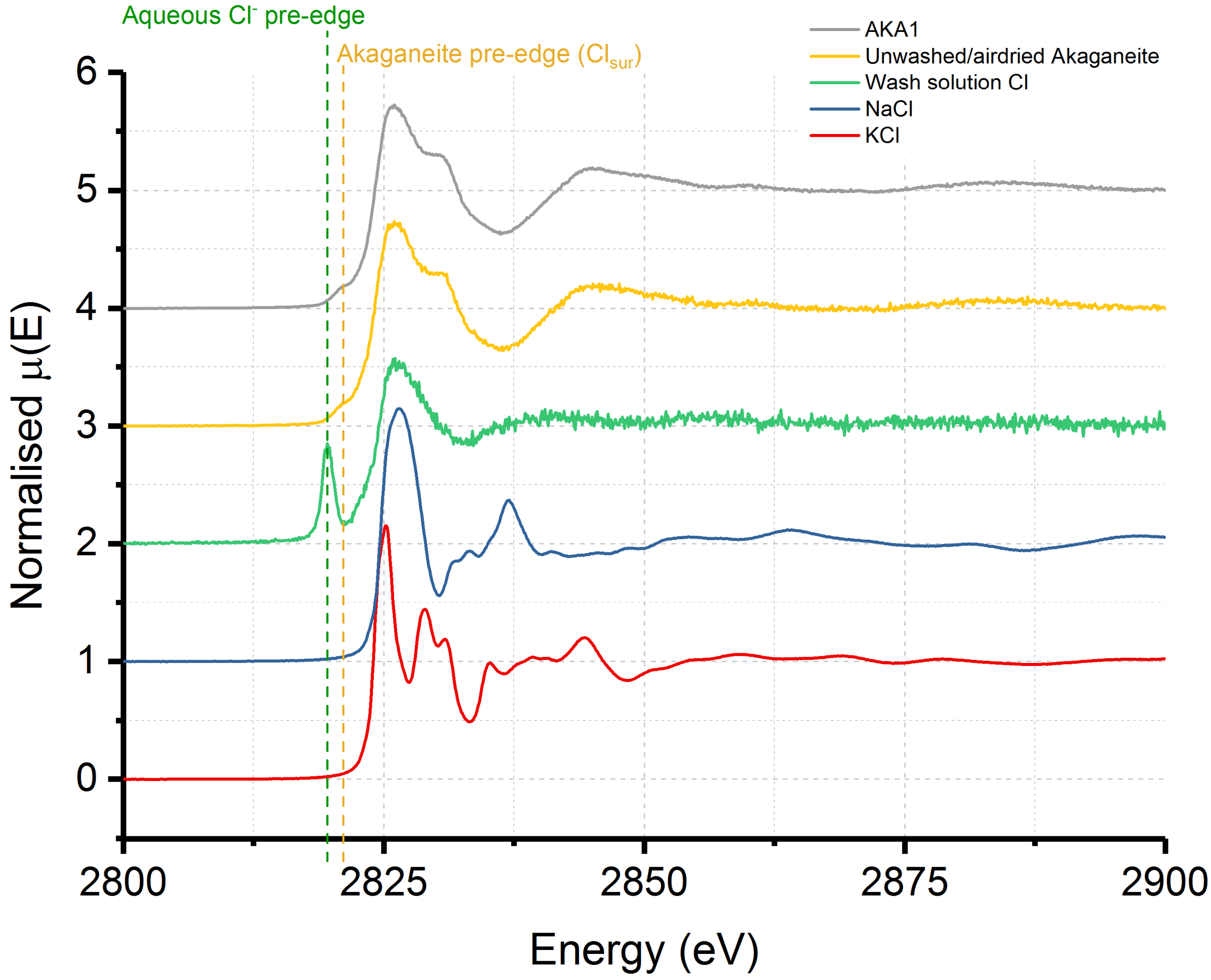

Fig. S6 Additional chlorine XAS standards, showing that the pre-edge feature of akaganeite is unaffected by heated drying at 50 °C, and does not arise from interaction of Cl^- with water

Fig. S7: Additional Cl XRF element maps

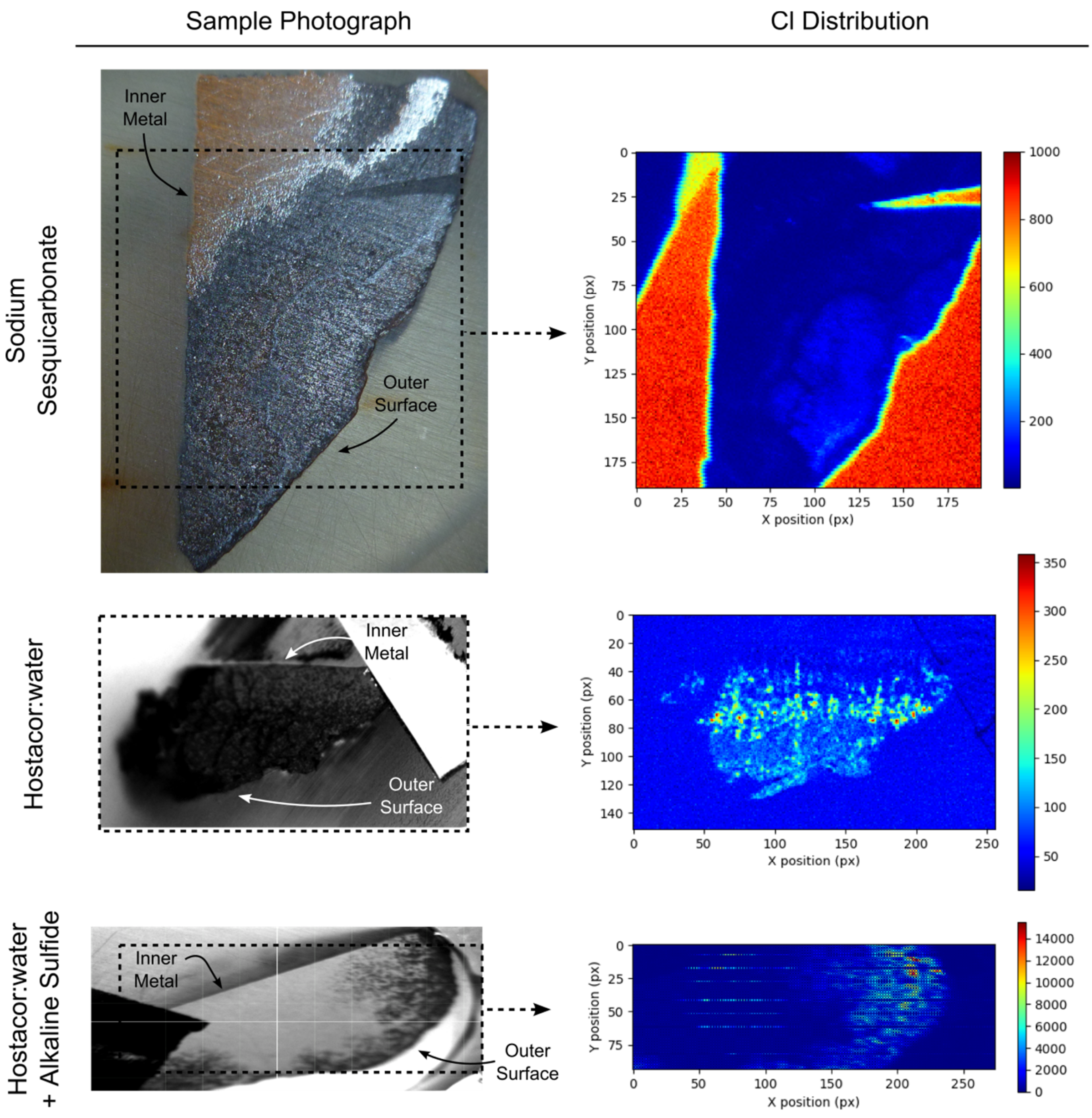

Fig. S7 Additional XRF elemental maps of the chlorine distribution in cut cross-sections from SS, HW and HWAS treated shot not included in the main document Fig. 3

Fig. S8: Additional peak-fit results

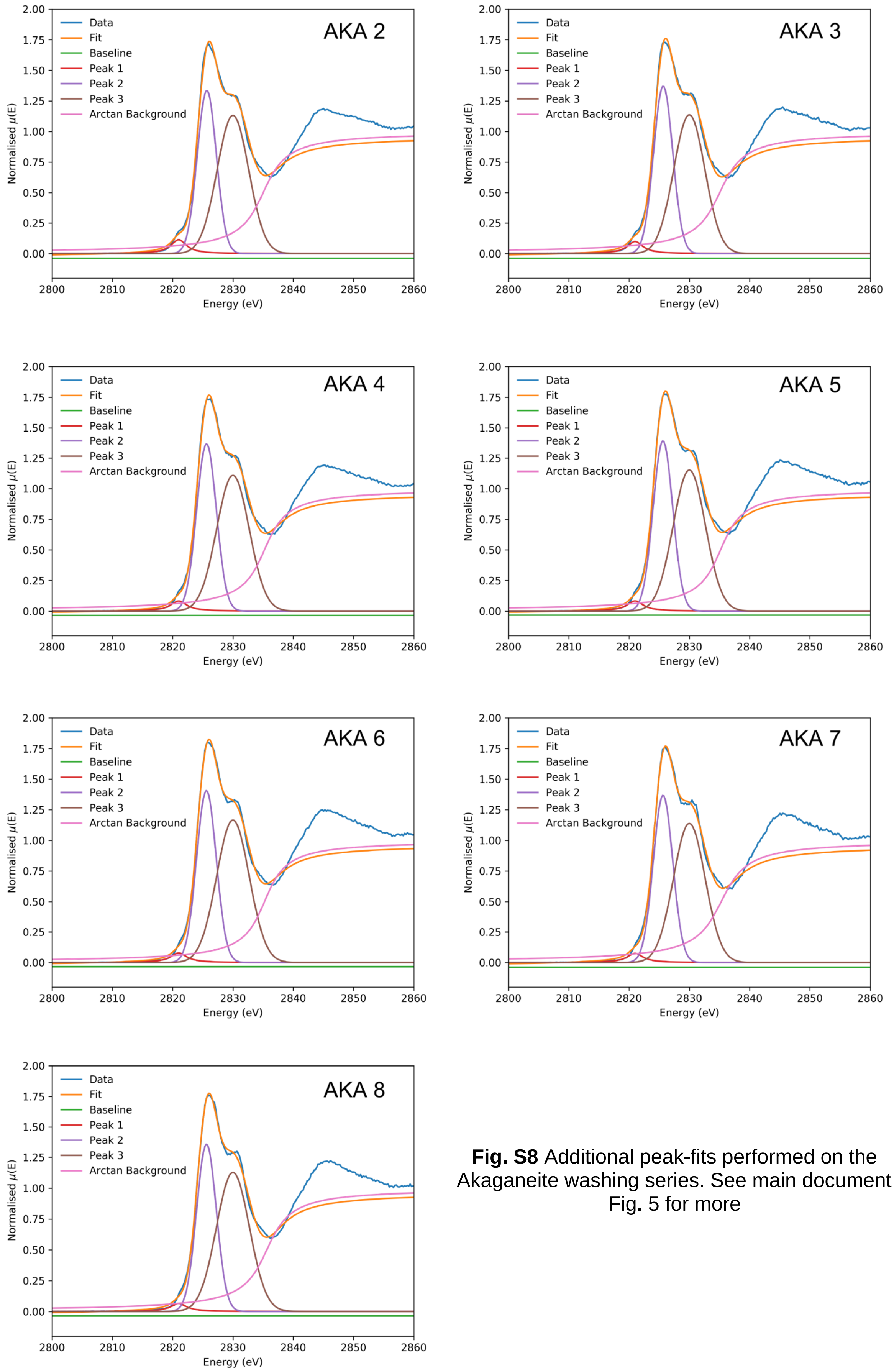

Fig. S8 Additional peak-fits performed on the Akaganeite washing series. See main document Fig. 5 for more

Fig. S9: Weight change since excavation

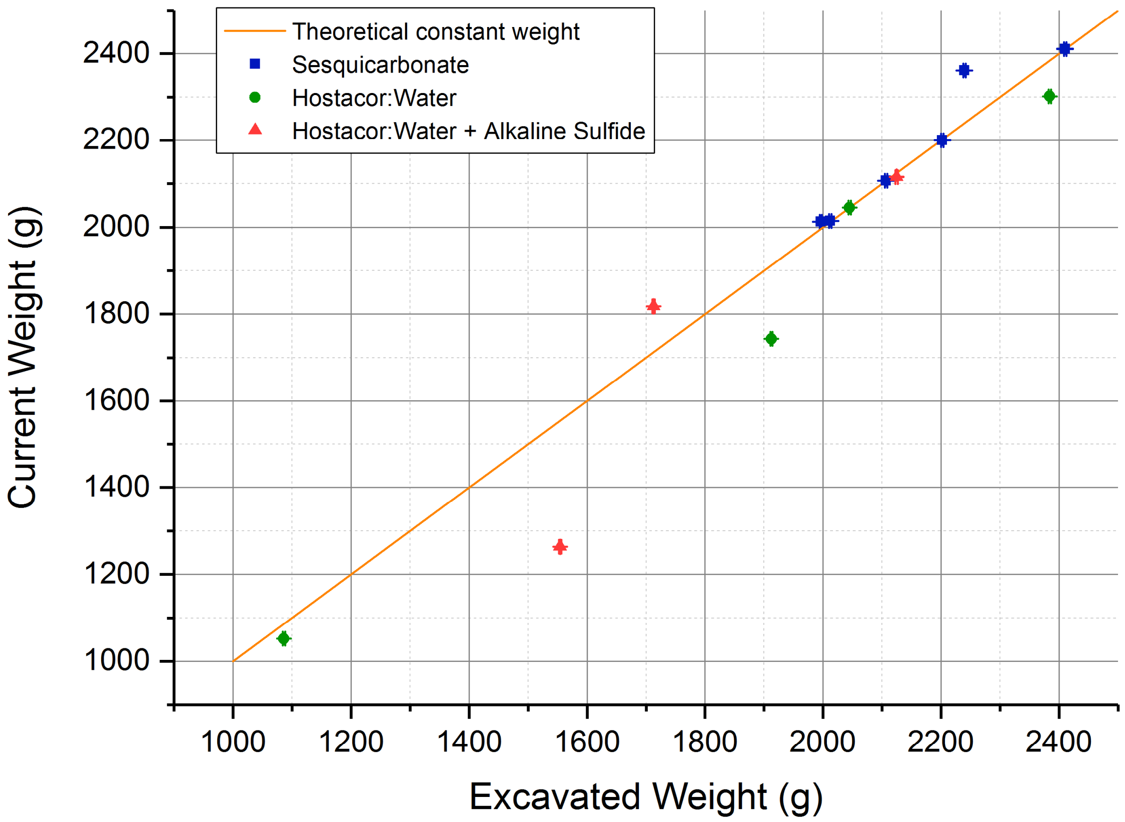

Fig. S9 Current (2017) measured weight of shot compared to recorded weight at excavation (1981-3)

SI References

[1] U. Schwertmann, R. M. Cornell, in *Iron Oxides*, Wiley VCH Verlag GmbH, Weinheim, Germany, **2003**, pp. 525–540.

[2] S. Reguer, F. Mirambet, E. Dooryhee, J. Hodeau, P. Dillmann, P. Lagarde, *Corros. Sci.* **2009**, *51*, 2795–2802.

[3] P. Refait, J. M. R. Génin, *Corros. Sci.* **1993**, *34*, 797–819.

[4] P. Refait, J. M. . Genin, *Corros. Sci.* **1997**, *39*, 539–553.

[5] S. P. Thompson, J. E. Parker, J. Potter, T. P. Hill, A. Birt, T. M. Cobb, F. Yuan, C. C. Tang, *Rev. Sci. Instrum.* **2009**, *80*, 75107.

[6] A. Altomare, N. Corriero, C. Cuocci, A. Falcicchio, A. Moliterni, R. Rizzi, *J. Appl. Crystallogr.* **2015**, *48*, 598–603.

[7] B. Ravel, M. Newville, *J. Synchrotron Radiat.* **2005**, *12*, 537–541.

[8] J. Anné, N. P. Edwards, A. van Veelen, V. M. Egerton, P. L. Manning, J. F. W. Mosselmans, S. Parry, W. I. Sellers, M. Buckley, R. A. Wogelius, *J. Anal. At. Spectrom.* **2017**, DOI 10.1039/C7JA00042A.
